# Association between brominated flame retardants (PBDEs and PBB153) exposure and hypertension in U.S. adults: results from NHANES 2005–2016

**DOI:** 10.1186/s12940-024-01103-0

**Published:** 2024-07-13

**Authors:** Dian Cheng, Zijun Chen, Jian Zhou, Yue Cao, Xin Xie, Yizhang Wu, Xiaorong Li, Xuecheng Wang, Jinbo Yu, Bing Yang

**Affiliations:** 1 Department of Cardiovascular Medicine, Shanghai East Hospital, School of Medicine, Tongji University, 150 Jimo Road, Shanghai, 200120 P.R. China; 2 School of Resources and Environmental Engineering, East China University of Science and Technology, 130 Meilong Road, Shanghai, 200237 P.R. China

**Keywords:** Brominated flame retardants, Hypertension, NHANES, Weighted quantile sum regression, Bayesian kernel machine regression

## Abstract

**Background:**

Brominated Flame Retardants (BFRs) have attracted widespread concern due to their environmental persistence and potential toxicity. This study aims to examine the association between BFRs exposure and hypertension.

**Methods:**

We used data from the National Health and Nutrition Examination Survey (NHANES) spanning 2005 to 2016 for the cross-sectional analysis. To evaluate the individual and combined impacts of BFRs exposure on hypertension, we utilized multivariate models, including generalized additive models, weighted quantile sum (WQS) regression, and Bayesian kernel machine regression (BKMR) models.

**Results:**

9882 individuals (48% male) aged ≥ 20 were included in the final analysis, of whom 4114 had hypertension. After controlling for potential covariates, higher serum concentrations of PBDE100 (OR: 1.26; 95% CI: 1.01, 1.57) and PBDE153 (OR: 1.50; 95% CI: 1.18, 1.88) were significantly associated with hypertension. A nonlinear relationship between PBDE28 and hypertension was observed (*P* = 0.03). Moreover, BFRs mixture were positively associated with the prevalence of hypertension in both the WQS (β:1.09; 95% CI: 1.02, 1.17; *P* = 0.02) and BKMR models.

**Conclusion:**

Our study suggested that BFRs exposure is positively associated with hypertension in the general population. To confirm this association and elucidate the mechanisms, further research is required.

**Supplementary Information:**

The online version contains supplementary material available at 10.1186/s12940-024-01103-0.

## Introduction

Brominated Flame Retardants (BFRs) are chemical compounds widely used in various products such as plastics, furniture, textiles, construction materials, and electrical and electronic devices to adhere to fire safety standards [1–3]. Due to the persistence, accumulation and environmental ubiquity, BFRs can be detected in wildlife around the world and in humans [4–6], even in newborns [7], which has raised considerable attention. Polybrominated diphenyl ethers (PBDEs) and polybrominated biphenyls (PBBs), as subgroups of BFRs, are considered more hazardous than other types [8]. PBDEs are classified into three principal varieties: penta-BDE, octa-BDE, and deca-BDE. Penta- and Octa-BDE were removed from the broad market in the United States in 2004, while the production and importation of deca-BDE ceased in 2013 [9]. The Stockholm Convention, an international treaty managed by the United Nations Environment Program, has categorized certain BFRs (such as PBDEs and PBBs) as persistent organic pollutants (POPs), leading to their restricted use and gradual phase-out [10]. Despite these restrictions, BFRs can still be consistently detected in consumer durables, foods and indoor dust. Prior research has revealed that BFRs can cause a range of harmful effects, including endocrine disruption, neurotoxicity, liver, and kidney damage, as well as negative impacts on reproduction and development, posing risks to the environment and human well-being [11–16]. Nevertheless, the cardiovascular implications of exposure to BFRs have not been thoroughly studied.

Hypertension, a prevalent cardiovascular disease (CVD) worldwide, impacts approximately 1.28 billion adults aged 30 to 79. In the United States, the age-adjusted prevalence of hypertension was 45.1% as of 2021 [17]. Elevated blood pressure is consistently associated with the development and progression of coronary artery disease, chronic renal disease, and stroke [18, 19]. Furthermore, hypertension frequently coexists with dyslipidemia, glucose intolerance, and type 2 diabetes, hence increasing the risk of CVD [20]. In addition to hereditary factors, hypertension is mainly affected by lifestyles, physical inactivity, psychological stress, and exposure to specific environmental contaminants [21–23]. There is increasing worry about environmental toxins, including heavy metals, air pollution, and POPs. Everett found that greater serum levels of polychlorinated biphenyl (PCB) 138 and PCB126 were associated with an elevated risk of hypertension, based on data from the National Health and Nutrition Examination Survey (NHANES) [24]. Valera discovered a direct correlation between PCB138 and hypertension risk among the Inuit population highly exposed to PCB138 [25]. While PBDEs and PCBs have comparable chemical structures and functional mechanisms, few studies have investigated the effect of BFRs exposure on hypertension in the general population.

Our study aims to delve the effect of BFRs exposure on hypertension by using the NHANES database. Additionally, we explore whether specific population subsets exhibit a more pronounced association between BFRs exposure and hypertension.

## Methods

### Study population

**Fig. 1:**
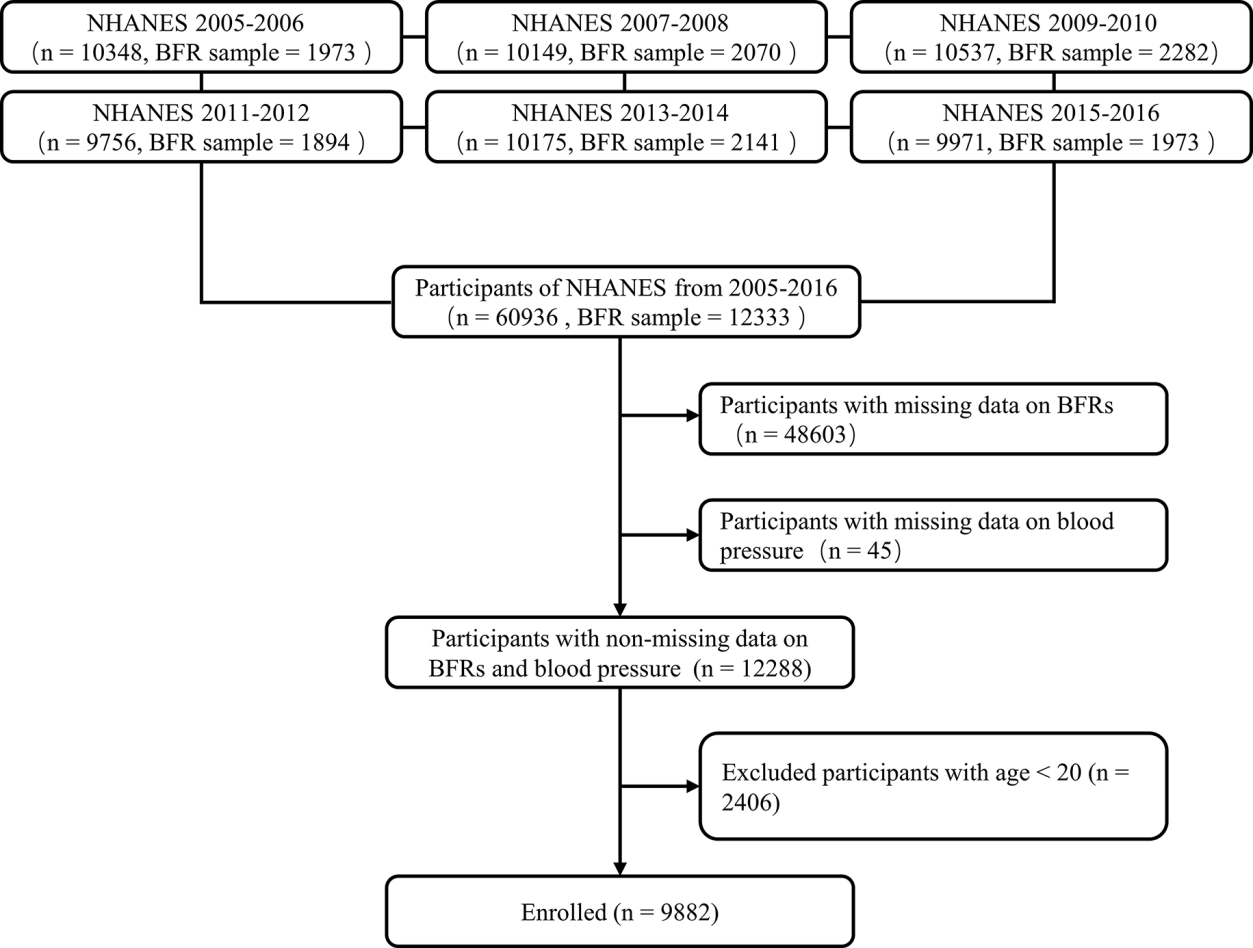
Flow chart of study participants. NHANES, National Health and Nutrition Examination Survey; BFRs, brominated flame retardants.

The data was obtained from NHANES, a nationwide cross-sectional survey conducted by the National Center for Health Statistics (NCHS) and the Centers for Disease Control and Prevention (CDC), to evaluate the health and nutritional conditions of the general U.S. population. The comprehensive survey design, methodologies, and data are available on the NHANES website (https://wwwn.cdc.gov/Nchs/Nhanes/2015-2016/BFRPOL_I.htm). The NHANES study protocol received approval from the NCHS research ethics review board, and participants gave written informed consent at enrollment. A total of 12,333 individuals from six consecutive cycles (NHANES 2005–2016), who underwent a series of serum BFRs measurements, were initially included. 45 subjects with missing data on blood pressure and 2406 subjects aged *<* 20 years were excluded. Finally, 9882 individuals were enrolled in the analyses (Fig. 1).

### Exposure variables

To avoid additional variability and potential bias introduced by lipid adjustment, we used serum BFR concentrations to reflect individual exposure levels [26, 27]. The quantification of PBB-153 and 11 PBDEs in serum was performed using automated liquid-liquid extraction and dilution gas chromatography high-resolution mass spectrometry. Table S1 illustrates the rates of detection and distribution of BFRs. To ensure the reliability of our study, we chose PBB153 and eight PBDEs with a detection rate over 65% as exposure factors. The eight PBDEs include 2,4,4´-Tribromodiphenyl ether (PBDE28), 2,2´,4,4´-Tetrabromodiphenyl ether (PBDE47), 2,2´,3,4,4´-Tetrabromodiphenyl ether (PBDE85), 2,2´,4,4´,5-Pentabromodiphenyl ether (PBDE99), 2,2´,4,4´,6-Pentabromodiphenyl ether (PBDE100), 2,2´,4,4´,5,5´-Hexabromodiphenyl ether (PBDE153), 2,2´,4,4´,5,6´-Hexabromodiphenyl ether (PBDE154), Decabromodiphenyl ether (PBDE209). Concentrations of serum BFRs below the lower limit of detection (LOD) were determined with the LOD value divided by the square root of 2.

### Outcome definition

In this study, the occurrence of hypertension is the primary outcome. During the personal interview addressing various health concerns, a standardized medical questionnaire was employed. Participants were queried, ‘Has a doctor or any medical professional ever informed you a diagnosis of high blood pressure or hypertension?’ Those who affirmed were categorized as hypertensive, while negative responses indicated an absence of hypertension. During each interview, the participants were also asked to report the medications that they have taken in the past 30 days. The blood pressures (BP) measurements of participants were taken by trained physicians in the mobile examination center (MEC). After five minutes of quiet sitting, participants take three consecutive BP readings to obtain the maximum inflation level (MIL). A fourth try is allowed in cases where the measurement is interrupted or incomplete. The average of the three or four BP recordings was calculated as systolic blood pressure (SBP) and diastolic blood pressure (DBP) for each participant. Hypertension was defined based on participants meeting at least one of the following criteria: (1) self-reported diagnosis of hypertension; (2) self-reported antihypertensive medication use; (3) SBP ≥ 140 mmHg and/or DBP ≥ 90 mmHg.

### Covariates

Based on previous literature [28–32], we incorporated a number of potentially confounding variables, including age, gender, race, education level, the family income to poverty ratio (PIR), serum cotinine, alcohol consumption, sleep disorders, depression, physical activity, dietary sodium intake, dietary potassium intake, body mass index (BMI), eGFR, history of diabetes and NHANES cycles (Figure S1). Demographic data, lifestyle information, disease history and physical measurements as well as laboratory tests were collected and administered by trained staffs according to standardized questionnaires and MEC. Demographic characteristics, including age (years) (20 ≤ Age < 65, Age ≥ 65), gender (male, female), race (Non-Hispanic White, Non-Hispanic Black, Mexican American, Other Race), education level (below high school, high school, above high school) and PIR (< 1, 1–3, > 3). PIR was calculated by dividing the yearly household income by the poverty threshold for the family size in the state of residence for that year, according to federal criteria. In this study, PIR was recoded as a dichotomous variable. Lifestyle included serum cotinine (≤ 1 µg/L, > 1 µg/L), alcohol consumption (< 12 drinks, ≥ 12 drinks), sleep disorders (yes or no), depression (yes or no), physical activity (activity or inactivity), dietary sodium intake (milligrams) and dietary potassium intake (milligrams). Serum cotinine levels indicate both active and passive smoking and was categorized into two groups (≤ 1 µg/L, > 1 µg/L) to distinguish between smokers and non-smokers [33]. Alcohol consumption was assessed by the question: “In any one year, have you had at least 12 drinks of any type of alcoholic beverage?” (yes/no). Sleeping disorders was evaluated with the question: “Have you ever informed a doctor or other health professional that you have difficulty sleeping?” (yes/no). Depression was tested using the Patient Health Questionnaire (PHQ-9), with total scores ranging from 0 to 27 points. A cutoff score of 10 was used to identify clinically relevant depression. Physical activity and inactivity were defined as less than four hours of moderate to high physical activities per week, according to the WHO Global Physical Activity Questionnaire [34]. Dietary sodium and potassium intake was extracted from 24-h dietary recall interviews in the NHANES database. BMI was calculated as weight (kg) /height (m^2^). We categorized participants into three BMI groups: normal (< 25 kg/m^2^), overweight (25–29.9 kg/m^2^), and obese (≥ 30 kg/m^2^). The estimated glomerular filtration rate (eGFR) was determined for each participant based on the Chronic Kidney Disease Epidemiology Collaboration (CKD-EPI) equation. Diabetes was defined as fasting plasma glucose (FPG) levels ≥ 7.0 mmol/L or/and glycosylated hemoglobin levels > 6.5%, self-reported diagnosis of diabetes or use of oral lowing glucose medications or insulin.

### Statistical analysis

Baseline characteristics were compared between participants with and without hypertension using the *t* and *χ2* test for continuous and categorical variables, respectively. Mean (standard deviation) or median with interquartile range (IQR) are used to display continuous values, while percentages are used for categorical variables. PBDEs and PBB-153 levels were log_10_ transformed to normalize the distribution. We applied the MEC weights according to NCHS guidance to account for the complex, multistage sampling design of the NHANES [35]. Weighted models were used in the multiple regression analyses.

We performed multivariate logistic regression analysis to assess the association between individual BFR and hypertension. Three models were applied, Model 1 was a crude model, Model 2 was controlled for age, race, and gender, and Model 3 was further controlled for education level, PIR, serum cotinine, alcohol consumption, sleep disorders, depression, physical activity, dietary sodium intake, dietary potassium intake, BMI, eGFR, history of diabetes and NHANES cycles based on Model 2. A generalized additive model investigated the nonlinear association between exposure to BFRs and hypertension. Wald χ^2^ tests were used to test for nonlinearity in the associations. Stratified analyses were performed by age, gender, education level, PIR, serum cotinine, alcohol consumption, sleep disorders, physical activity, BMI and history of diabetes. Interaction tests were also carried out to assess the individualized effects of BFRs on hypertension across different subgroups in the multivariate logistic regression model.

To investigate the effect of exposure to BFRs mixture on hypertension, we used the quantile weighted quantile sum (WQS) regression and Bayesian kernel machine regression (BKMR) analysis. Using a weighted index, in which the relative importance of each predictor variable establishes its total impact, the WQS regression assesses the combined impact of all predictor factors on the result. An estimation set comprising 40% of the training data and a validation set comprising 60% of the data were generated randomly. Bootstrap resampling, with 1000 iterations, facilitated the estimation of model parameters [36]. The BKMR provides succinct and versatile estimations of the multivariate exposure-response function [37, 38]. The integrative effect of the mixed BFRs on hypertension was determined by estimating the different risk of hypertension, when all 9 BFRs were maintained at the 10th to 90th percentiles (in increments of 10th percentile points) as compared to their 50th percentile. Moreover, we calculated the posterior inclusion probability (PIP) for each BRF in mixtures to determine the BRF that contributed most to the prevalence of hypertension. Following full covariate adjustment, this model underwent 10,000 iterations using Markov Chain Monte Carlo.

The statistical analyses were conducted using R software (version 3.6.0). A two-sided *P* value < 0.05 was considered statistically significant.

## Results

### Baseline characteristics

There were 4,114 cases of hypertension among the 9,882 participants in the study. The categories of hypertension diagnosis were listed in Table S2. Compared to those without hypertension, participants with hypertension were older, had a higher proportion of males, non-Hispanics, less educated, and prevalence of diabetes. PIR, sleep disorders, depression, physical activity, dietary sodium and potassium intake also differed between the two groups (*P* < 0.01). In addition, participants with hypertension have higher BMI and lower eGFR levels (Table 1). Participants with hypertension showed higher BFRs concentrations than those without hypertension (Table S3).

**Table 1:** Characteristics of the study population

Variable	Overall (*n* = 9882)				Non-hypertension (*n* = 5768)	Hypertension (*n* = 4114)	*P* value
Age, years	49.30 (17.87)				41.9 (15.88)	59.66 (15.15)	< 0.01**
< 65, n (%)	7558 (76.48)				5152 (89.32)	2436 (58.78)	
≥ 65,n (%)	2324 (23.51)				616 (10.68)	1708 (41.52)	
Gender, n (%)							0.02*
Female	5120 (51.81)				3048 (52.84)	2072 (50.36)	
Male	4762 (48.19)				2720 (47.16)	2042 (49.64)	
Race, n (%)							< 0.01**
Non-Hispanic White	4219 (42.69)				2422 (41.99)	1797 (43.68)	
Non-Hispanic Black	2048 (20.72)				996 (17.27)	1052 (25.57)	
Mexican American	1602 (16.21)				1048 (18.17)	554 (13.47)	
Other Race	2013 (20.37)				1302 (22.57)	711 (17.28)	
Education level, n (%)							< 0.01**
Below high school	2588 (26.19)				1340 (23.23)	1248 (30.34)	
High school	5080 (51.41)				1246 (21.60)	968 (23.53)	
Above high school	2214 (22.40)				3182 (55.17)	1898 (46.14)	
PIR, n (%)							< 0.01**
< 1	2138 (21.64)				1239 (21.48)	899 (21.85)	
1 ~ 3	3564 (36.07)				2355 (40.83)	1825 (44.36)	
> 3	4180 (42.30)				2174 (37.69)	1390 (33.79)	
Serum cotinine, µg/L	54.75 (124.29)				55.31 (121.85)	53.98 (127.64)	0.38
≤ 1 µg/L, n (%)	7040 (71.24)				2926 (50.73)	2338 (56.83)	
> 1 µg/L, n (%)	2842 (28.76)				2842 (49.27)	1776 (43.17)	
Alcohol consumption, n (%)							0.09
< 12 drinks	4218 (42.68)				2420 (41.96)	1798 (43.70)	
≥ 12 drinks	5664 (57.32)				3348 (58.04)	2316 (56.30)	
Sleep disorders, n (%)							< 0.01**
Yes	2478 (25.08)				1156 (20.04)	1322 (32.13)	
No	7404 (74.92)				4612 (79.96)	2792 (67.87)	
Depression, n (%)							< 0.01**
Yes	801 (8.11)				394 (6.83)	407 (9.89)	
No	9081 (91.89)				5374 (93.17)	3707 (90.11)	
Physical activity, n (%)							< 0.01**
Activity	7356 (74.44)				4573 (79.28)	2783 (67.65)	
Inactivity	2526 (25.56)				1195 (20.72)	1331 (32.35)	
Dietary sodium, mg	1675.47 (670.24)				1720.99 (685.13)	1611.65 (643.47)	< 0.01**
Dietary potassium, mg	1303.27 (483.32)				1322.09 (487.03)	1276.88 (476.87)	< 0.01**
BMI, Kg/m^2^	29.09 (6.71)				27.94 (6.22)	30.69 (7.04)	< 0.01**
< 25 Kg/m^2^, n (%)	2835 (28.69)				2017 (34.97)	818 (19.88)	
25-29.9 Kg/m^2^, n (%)		3721 (37.65)			2011 (34.86)	1981 (48.15)	
≥ 30 Kg/m^2^, n (%)	3326 (33.66)				1740 (30.17)	1315 (31.96)	
eGFR, mL/min	44.46 (48.79)				46.85 (51.46)	41.12 (44.56)	< 0.01**
Systolic blood pressure, mmHg			123.85 (18.55)		115.33 (10.98)	135.69 (20.20)	< 0.01**
Diastolic blood pressure, mmHg		69.82 (12.99)			68.39 (10.71)	71.82 (15.43)	< 0.01**
Diabetes, n (%)							< 0.01**
Yes	1467 (14.85)				383 (6.64)	1084 (26.35)	
No	8415 (85.15)				5385 (93.36)	3030 (73.65)	
NHANES cycles, n (%)							0.02*
2005–2006	1348 (13.64)				834 (14.46)	514 (12.49)	
2007–2008	1744 (17.65)				1016 (17.61)	728 (17.70)	
2009–2010	1869 (18.91)				1101 (19.09)	768 (18.67)	
2011–2012	1552 (15.71)				920 (15.95)	632 (15.36)	
2013–2014	1740 (17.61)				983 (17.04)	757 (18.40)	
2015–2016	1629 (16.48)				914 (15.85)	715 (17.38)	

PIR, the family income to poverty ratio; BMI, body mass index; eGFR, estimated Glomerular Filtration Rate; NHANES, National Health and Nutrition Examination Survey. *p < 0.05, **p < 0.01

### Associations between individual BFRs and hypertension

The multivariable logistic regression analysis revealed that all BFRs except PBDE209 were significantly positively associated with hypertension in model 1. After adjusting for demographic characteristics and all covariates, PBDE100 and PBDE153 were still significantly associated with hypertension, exhibiting 26% and 50% increase in odds (95% CI: 1.01, 1.57; *P* = 0.04; 95% CI: 1.18 1.88; *P* < 0.01; Table 2), respectively.

**Table 2:** Multivariable logistic regression analysis of log-transformed serum BFRs with hypertension

Variable	Model 1			Model 2			Model 3		
	OR	95%CI	*P* value	OR	95%CI	*P* value	OR	95%CI	*P* value
lgPBDE28	3.04	2.46–3.75	< 0.01**	1.22	0.92–1.61	0.16	1.24	0.92, 1.69	0.16
lgPBDE47	1.88	1.55–2.29	< 0.01**	1.14	0.91–1.43	0.26	1.13	0.89, 1.43	0.30
lgPBDE85	1.67	1.41–1.97	< 0.01**	1.18	0.97–1.42	0.09	1.14	0.93, 1.39	0.20
lgPBDE99	1.58	1.33–1.88	< 0.01**	1.06	0.87–1.30	0.54	1.03	0.84, 1.26	0.76
lgPBDE100	1.78	1.48–2.15	< 0.01**	1.25	1.02–1.55	0.04*	1.26	1.01–1.57	0.04*
lgPBDE153	1.45	1.18–1.79	< 0.01**	1.47	1.17–1.85	<0.01**	1.50	1.18–1.88	<0.01**
lgPBDE154	1.67	1.40–2.01	< 0.01**	1.13	0.92–1.39	0.23	1.13	0.91, 1.42	0.27
lgPBDE209	1.16	0.88–1.53	0.30	1.11	0.81–1.50	0.51	1.04	0.76, 1.43	0.78
lgPBB153	3.27	2.84–3.77	< 0.01**	1.10	0.93–1.31	0.25	1.03	0.87–1.23	0.71

Model 1: crude model

Model 2: adjusted for age, gender, race

Model 3: adjusted for age, gender, race, education level, PIR, serum cotinine, alcohol consumption, sleep disorders, depression, physical activity, dietary sodium intake, dietary potassium intake, BMI, eGFR, history of diabetes and NHANES cycles

lg, log-transformed; OR, odd ratio; CI, confidence interval. *p < 0.05, **p < 0.01

**Fig. 2:**
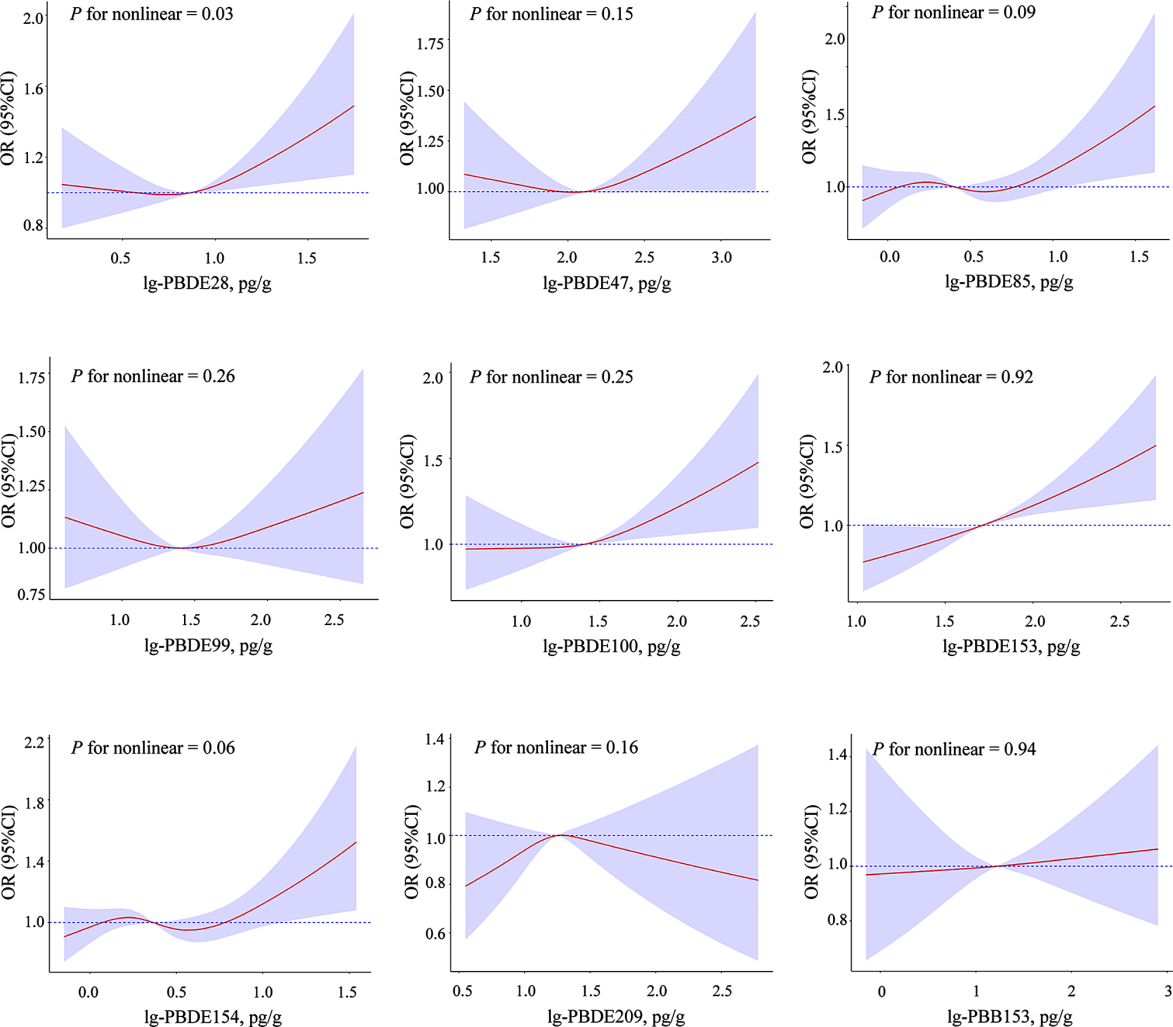
Cubic splines for the associations of log-transformed serum BFRs with Hypertension. The horizontal dashed line represents the OR = 1.00. The red lines indicate multivariate-adjusted OR and the purple shaded area represents the 95%CI. All models were adjusted for age, gender, race, education level, PIR, serum cotinine, alcohol consumption, sleep disorders, depression, physical activity, dietary sodium intake, dietary potassium intake, BMI, eGFR, history of diabetes and NHANES cycles. lg, log-transformed; OR, odd ratio; CI, confidence interval

The generalized additive models revealed a non-linear relationship between PBDE28 and hypertension (*P* for nonlinear = 0.03). Non-linear relationships were not significantly observed for the remaining eight BFRs (*P* for non-linearity > 0.05) (Fig. 2).

### Stratified associations between BFRs and hypertension

As presented in Table 3, Interaction analyses demonstrated that PBB153 had obvious interaction with age and PBDE28 interacted with BMI. PBB153 was significantly associated with hypertension in participants with age < 65 (OR: 2.38; 95% CI: 2.05, 2.76; *P* < 0.001; *P* for interaction < 0.001), while PBDE28 (OR: 1.54; 95% CI: 1.04, 2.27; *P* = 0.03; *P* for interaction = 0.05) showed significantly association for hypertension in participants with BMI < 30. However, no significant differences were observed between subgroups stratified by gender, education level, PIR, serum cotinine, alcohol consumption, sleep disorders, physical activity and history of diabetes (Table S4).

**Table 3:** Association between log-transformed serum BFRs and hypertension in subgroups stratified by age and BMI

Age	Age < 65				Age ≥ 65					*P*-int
	OR	CI	*P* value		OR		CI	*P* value		
lgPBDE28	2.40	1.73–3.32	<0.01**		1.31		0.75–2.28	0.34		0.53
lgPBDE47	1.35	1.02–1.8	0.04*		1.29		0.81–2.06	0.28		0.51
lgPBDE85	1.30	1.02–1.65	0.04*		1.21		0.79–1.84	0.38		0.80
lgPBDE99	1.17	0.92–1.48	0.21		1.15		0.78–1.69	0.49		0.46
lgPBDE100	1.41	1.07–1.86	0.02*		1.37		0.89–2.09	0.15		0.67
lgPBDE153	1.44	1.06–1.94	0.02*		1.83		1.23–2.72	<0.01**		0.96
lgPBDE154	1.31	1.00-1.71	0.06		1.18		0.78–1.79	0.43		0.64
lgPBDE209	1.19	0.84–1.69	0.33		1.12		0.65–1.93	0.69		0.86
lgPBB153	2.38	2.05–2.76	<0.01**		1.06		0.72–1.55	0.77		< 0.01**
BMI	BMI < 30				BMI ≥ 30					*P-int*
	OR	CI		*P* value	OR	CI			*P* value	
lgPBDE28	1.54	1.04–2.27		0.03*	0.87	0.59–1.3			0.50	0.05*
lgPBDE47	1.33	0.98–1.80		0.07	0.89	0.63–1.24			0.48	0.08
lgPBDE85	1.28	0.97–1.69		0.08	0.95	0.7–1.29			0.74	0.15
lgPBDE99	1.17	0.89–1.54		0.26	0.86	0.64–1.15			0.30	0.11
lgPBDE100	1.36	1.02–1.82		0.04*	1.11	0.81–1.53			0.51	0.36
lgPBDE153	1.33	1.01–1.75		0.04*	1.89	1.37–2.61			<0.01**	0.07
lgPBDE154	1.28	0.95–1.72		0.10	0.94	0.68–1.32			0.74	0.18
lgPBDE209	1.16	0.77–1.76		0.47	0.98	0.59–1.60			0.92	0.75
lgPBB153	1.18	0.94–1.47		0.16	0.91	0.68–1.22			0.53	0.06

BMI, body mass index; lg, log-transformed; OR, odd ratio; CI, confidence interval

*p < 0.05, **p < 0.01

### Association between all BFRs and hypertension in WQS model

**Fig. 3:**
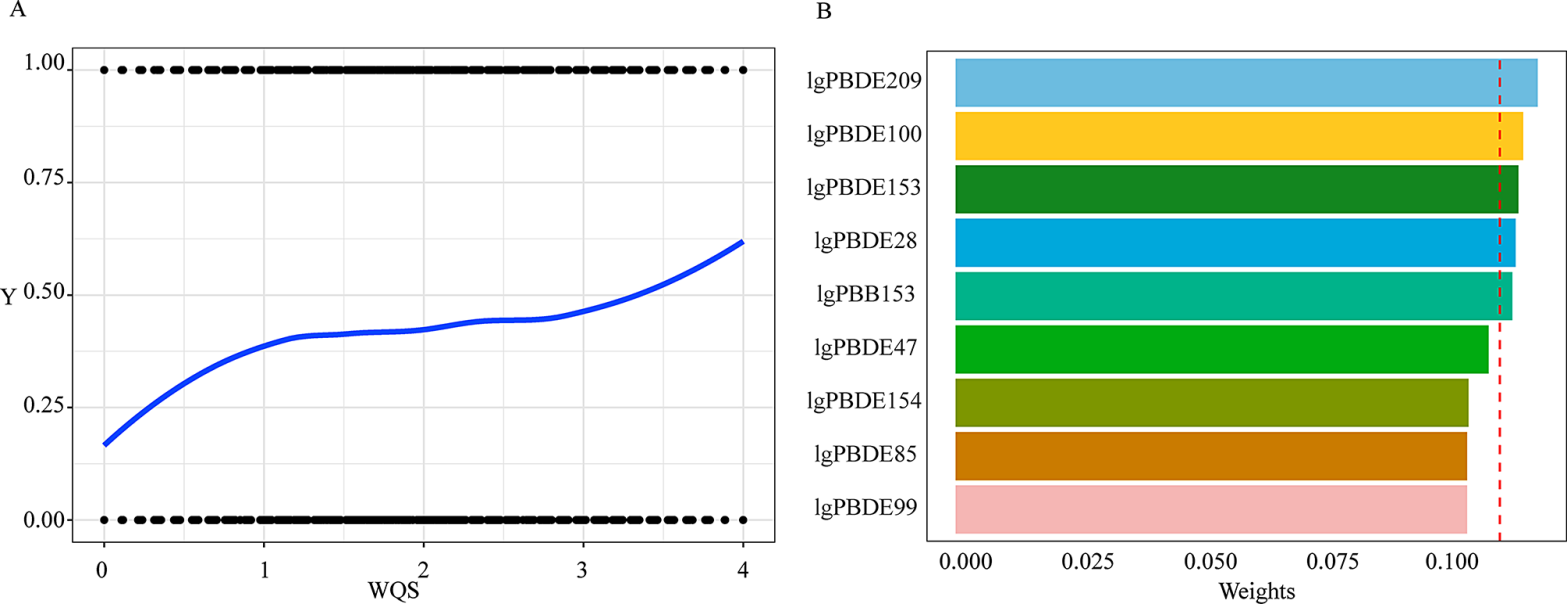
Association between BFRs exposure and hypertension by WQS model. (**A**) The combined effects of mixed exposure to BFRs. (**B**) The weights of each BFR for hypertension in positive direction. The dashed red line represents the cutoff value (by default equal to the inverse of the number of elements in the mixture). The model was adjusted for age, gender, race, education level, PIR, serum cotinine, alcohol consumption, sleep disorders, depression, physical activity, dietary sodium intake, dietary potassium intake, BMI, eGFR, history of diabetes and NHANES cycles. WQS, weighted quantile sum; lg, log-transformed

WQS, weighted quantile sum; lg, log-transformed.

The multiple exposure effects of BFRs on hypertension were investigated using the WQS model. After accounting for all potential covariates, the WQS model identified a positive association between exposure to BFRs mixture and the prevalence of hypertension (β: 1.09; 95% CI: 1.02, 1.17; *P* = 0.02) (Fig. 3A; Table 4). Figure 3B presented that PBDE209 contributed the most to the WQS index, followed by PBDE100, PBDE153, PBDE28 and PBB153 were relatively important for hypertension due to their higher calculated weights. The WQS regression in the negative direction showed no significant association of the BFRs mixture with hypertension (β: 1.09; 95% CI: 0.99, 1.19; *P* = 0.07) (Table 4).

**Table 4:** Association between exposure to BFRs mixture and hypertension by WQS model

Model	β	95%CI	*P* value
WQS			
Positive	1.09	1.02–1.17	0.02**
Negative	1.09	0.99–1.19	0.07

Model was adjusted for age, gender, race, education level, PIR, serum cotinine, alcohol consumption, sleep disorders, depression, physical activity, dietary sodium intake, dietary potassium intake, BMI, eGFR, history of diabetes and NHANES cycles

WQS, weighted quantile sum; CI, confidence interval. *p < 0.05

### Association between all BFRs and hypertension in BKMR model

**Fig. 4:**
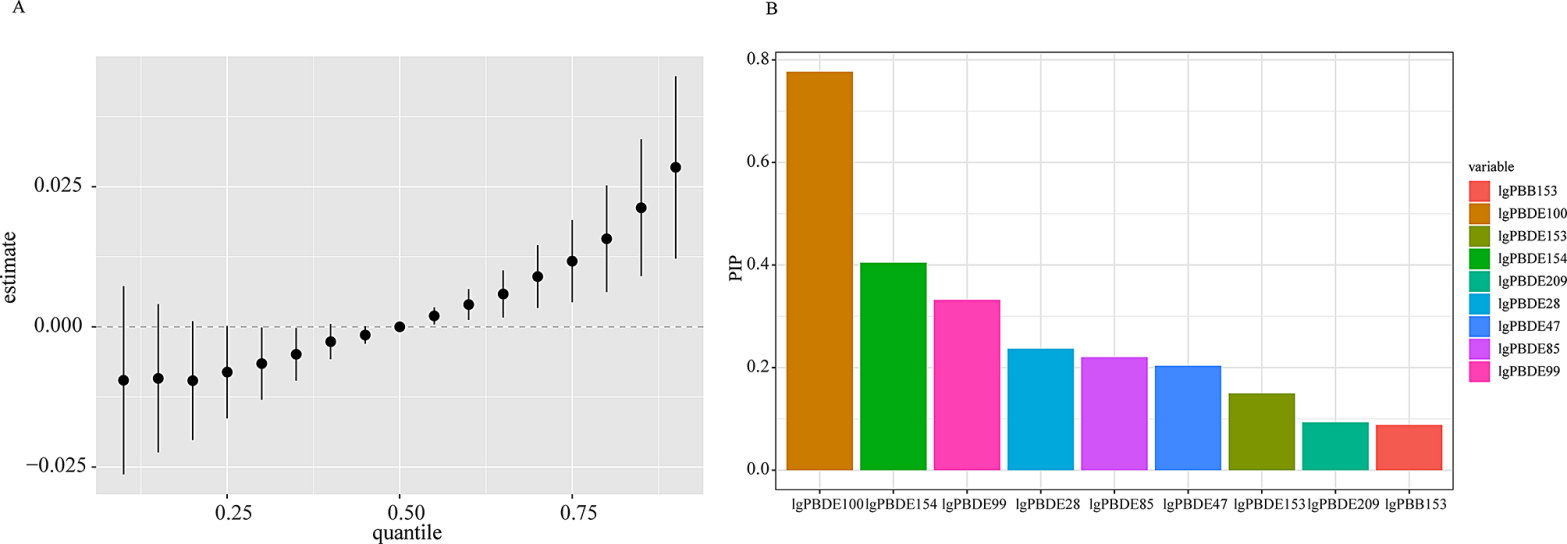
Association between combined BFRs exposure and hypertension analyzed by BKMR model. (**A**) Overall effects of BFRs mixture on hypertension at all concentrations ranged from the first quantile (10%) to the third quantile (90%) relative to the median (50%) level. (**B**) Posterior inclusion probabilities (PIPs) of each BFR for hypertension. Adjusted for age, gender, race, education level, PIR, serum cotinine, alcohol consumption, sleep disorders, depression, physical activity, dietary sodium intake, dietary potassium intake, BMI, eGFR, history of diabetes and NHANES cycles

In the BKMR model, Fig. S2 summarizes the results of the univariate exposure–response functions with other concentrations fixed at the median. A significant positive trend was observed between PBDE100 and hypertension. As shown in Fig. 4A, a significant overall association between exposure to BFRs mixture and increased risk of hypertension was observed when the mixture was at the 55th percentile or above, compared to their 50th percentile. Among BFRs mixtures, the highest PIPs for hypertension were estimated for PBBDE100 (PIP = 0.78) and PBDE154 (PIP = 0.40) (Fig. 4B). Meanwhile, the interaction between BFRs were not significant (Fig. S3).

## Discussion

In this study, various statistical approaches were employed to thoroughly evaluate the effects of both individual and mixed BFRs exposures on hypertension. Our research demonstrated that PBDE100 and PBDE153 were independently associated with the prevalence of hypertension after accounting for potential covariates. A nonlinear relationship between PBDE28 and hypertension was observed. Positive overall trends between BFRs mixture and hypertension were found in both the WQS and BKMR models.

Epidemiological studies on the relationship between BFRs exposure and hypertension are extremely sporadic. Smarr et al. reported an elevated odds of gestational hypertension associated with PBDE66, though this finding was not statistically significant [39]. Eslami et al. showed a significant correlation between total PBDEs and pre-eclampsia in first-time moms [40]. However, these studies focused on pregnant populations and did not investigate the correlation between PBDEs and hypertension in the general population. In contrast, our study indicated a positive association between BFRs and hypertension in general adults. Che et al. examined the association between BFRs and metabolic syndrome, including its components, using community-based data. Their research found no significant association between exposure to BFRs and hypertension [41]. The sample size of the study (4641 adults) was substantial for epidemiological research, but many cases were excluded for not meeting the inclusion criteria during the study period. Our study had a larger sample size, potentially increasing the statistical power and the ability to detect associations. The definition and determination of hypertension are consistent across both studies, as are the PBDEs and PBBs involved. However, variations in study periods may lead to differences in detection frequencies and levels of specific congeners. Additionally, our study included more covariates, whereas their study included fewer and different covariates, which may lead to residual confounding. Differences in population characteristics, covariate selection, and study periods may all contribute to the differing results. Our study included adults from six consecutive cycles from the NHANES database to evaluate the association between exposure to BFRs and hypertension. In the individual BFR analysis, PBDE100 and PBDE153 were significantly associated with hypertension. Meanwhile, we employed the WQS regression and BKMR models, two statistical tools, to examine the complexity of BFRs mixture exposure, avoiding the biases of traditional methods, which may simulate single chemical without considering the potential collinearity among similar compounds. To mitigate the effect of extreme concentrations and to evaluate the total risk of chemical exposure, WQS regression quantile-sorts continuous variables using bootstrap sample weights. In our study, the results from the WQS analysis indicate a significant positive association between mixed BFRs exposure and hypertension. Simultaneously, both the univariate exposure-response analysis and the combined exposure analysis using the BKMR model suggest that certain individual BFRs and mixed BFRs may exhibit a positive relationship with hypertension. In the WQS analysis, PBDE209 contributes the most to the WQS index, followed by PBDE100 and PBDE153, which have relatively greater impacts on hypertension. Meanwhile, in the BKMR model, PBDE100 has the highest PIPs. In the multivariable regression analysis, several PBDEs and PBB153 are significantly associated with hypertension in the crude model. Even after adjusting for all variables, PBDE100 and PBDE153 remain show significant association with hypertension. Across multiple statistical strategies applied in this study, PBDE100 consistently demonstrates a significant association with hypertension in both individual and mixed BFRs exposures.

Although the potential mechanisms by which BFRs exposure affects blood pressure are unclear, several hypotheses could be considered. First, evidence indicates that human exposure to certain POPs can disrupt lipid homeostasis, trigger diabetes, promote obesity, and related diseases [42–44], all of which are common conditions in hypertensive patients [45, 46]. Current biological evidence suggests that PBDEs may contribute to the development of gestational diabetes mellitus (GDM), with PBDE-154 being correlated with an increased risk of GDM [47]. GDM and type 2 diabetes are clinical manifestations of the same entity, both attributed to insulin resistance, which is a known precipitant of cardiovascular diseases including hypertension [48, 49]. Therefore, the link between POPs and hypertension may be due to the increases in dyslipidemia, diabetes, and/or obesity caused by POPs. Simultaneously, previous research has demonstrated that octa- and deca-BDEs can cause degenerative alterations and kidney histopathology in rats [50]. Perinatal exposure to PBDE mixtures (DE-71) in rats disrupts blood pressure homeostasis in later adulthood, which may partly result from toxic effects on the kidneys and renal pathology, leading to excessive sodium retention and hypertension [51]. Additionally, previous studies discovered that HepG2 cells, wild-type N2 worms, and adipocytes are susceptible to oxidative stress induced by PBDE-47 at different concentrations [15, 52]. The presence of reactive oxygen species, a hallmark of oxidative stress, was demonstrated in human umbilical vein endothelial cells exposed to PBDE-209 [53]. Another study based on NHANES data from 2007 to 2016 analyzed the relationship between BFR levels and oxidative stress markers in American adults, revealed a positive association of BFRs exposure with oxidative stress markers. According to clinical and experimental findings, hypertension is linked to inflammation and immune cell activation, which are mostly caused by oxidative stress [54]. Overall, we speculate that, BFRs, as a unique exogenous chemical, could influence blood pressure levels by affecting endocrine hormones, renal function, oxidative stress, and metabolic pathways. Prospective cohort studies and in vitro/vivo experimental research are needed to confirm and examine the precise relationship and underlying mechanisms between BFRs exposure and hypertension.

Our study possesses certain advantages: It is the first to examine the relationship between BFRs and hypertension in a nationally representative sample of US adults. This study employed generalized additive regression, WQS regression, and BKMR models to comprehensively investigate the impacts of exposure to individual and overall BFRs on hypertension. Nevertheless, this study has certain constraints. Firstly, the observational study methodology makes it impossible to establish any causal links based on this data. Secondly, there is currently no consensus on the optimal method for measuring lipophilic chemicals in serum. This study used serum BFR concentrations to indicate exposure levels, which may not fully represent individual exposure and could lead to measurement errors. Thirdly, despite frequently using covariates in regression models, it is important to acknowledge the potential influence of unmeasured confounding effects. Fourthly, the pathogenic properties and mechanisms of BFRs in animals and humans are unclear. Besides, since our study participants were American adults, the applicability of our findings to other populations remains uncertain. Future longitudinal studies are needed to establish a causal relationship between exposure to BFRs and hypertension and verify the findings in more extensive populations. Moreover, experimental exploration of the specific biological mechanisms of BFRs represents an important direction for future studies.

## Conclusion

In summary, our investigation showed a significant positive association between BFRs exposure and hypertension in the general adults. This would help enhance public awareness about preventing exposure to BFRs. Further research would be required to confirm our findings and elucidate the potential mechanisms.

### Electronic supplementary material

Below is the link to the electronic supplementary material.

Supplementary Material 1

## Data Availability

No datasets were generated or analysed during the current study.

## Abbreviations

BFRs: Brominated flame retardants
PBDEs: Polybrominated diphenyl ethers
PBBs: Polybrominated biphenyls
POPs: Persistent organic pollutants
CVD: Cardiovascular disease
PCB: Polychlorinated biphenyl
NHANES: National health and nutrition examination survey
MEC: Mobile examination center
SBP: Systolic blood pressure
DBP: Diastolic blood pressure
PIR: The family income to poverty ratio
WQS: Weighted quantile sum
BKMR: Bayesian kernel machine regression
BMI: Body mass index
eGFR: Estimated glomerular filtration rate
GDM: Gestational diabetes mellitus
