# Supplementary material for: Association between brominated flame retardants (PBDEs and PBB153) exposure and hypertension in U.S. adults: results from NHANES 2005–2016
Source: Environ Health. 2024 Jul 13;23:64. doi: 10.1186/s12940-024-01103-0 (PMC11245865; doi:10.1186/s12940-024-01103-0)
Supplement: Supplementary file 1 — Supplementary Material 1 [file 12940_2024_1103_MOESM1_ESM.docx]

**Table S1. The detection rate of the BFRs in NHANES 2005-2016.**

| Exposures | The number of participants with BFRS at or above LOD | The number of participants with BFRS below LOD | Detection rate (%) |
| --- | --- | --- | --- |
| PBDE17 | 799 | 11574 | 6.46 |
| PBDE28 | 12035 | 378 | 96.95 |
| PBDE47 | 12421 | 0 | 100.00 |
| PBDE66 | 1734 | 10687 | 13.96 |
| PBDE85 | 9655 | 2766 | 77.73 |
| PBDE99 | 12421 | 0 | 100.00 |
| PBDE100 | 12421 | 0 | 100.00 |
| PBDE153 | 12421 | 0 | 100.00 |
| PBDE154 | 9687 | 2718 | 78.09 |
| PBDE183 | 5315 | 7106 | 42.79 |
| PBDE209 | 8598 | 3799 | 69.36 |
| PBB153 | 11115 | 1298 | 89.54 |

**Table S2. The categories of hypertension diagnosis.**

| **Category** | **Number of Patients** |
| --- | --- |
| Self-reported hypertension | 3516 (85.5%) |
| Self-reported antihypertensive medication use | 3044 (74%) |
| SBP ≥ 140 mmHg and/or DBP ≥ 90 mmHg | 1752 (42.6%) |
| Self-reported hypertension and medication use | 3044 (74%) |
| Self-reported hypertension and BP values | 1154 (28.1%) |
| Self-reported medication use and BP values | 1043 (25.4%) |
| Self-reported hypertension, medication use and BP values | 1043 (25.4%) |

**Table S3. Characteristics of the study population.**

| Variable | Overall  (n=9882) | Non-hypertension (n=5768) | Hypertension  (n=4114) | *P* value |
| --- | --- | --- | --- | --- |
| PBDE28, pg/g | 9.07 (6.66) | 8.34 (5.98) | 10.09 (7.39) | <0.01** |
| PBDE47, pg/g | 173.35 (168.12) | 162.11 (153.38) | 189.11 (185.72) | <0.01** |
| PBDE85, pg/g | 3.87 (4.76) | 3.60 (4.14) | 4.26 (5.48) | <0.01** |
| PBDE99, pg/g | 38.62 (49.49) | 36.17 (46.56) | 42.07 (53.15) | <0.01** |
| PBDE100, pg/g | 35.89 (35.88) | 33.49 (31.07) | 39.24 (41.46) | <0.01** |
| PBDE153, pg/g | 71.51 (63.63) | 68.63 (57.66) | 75.55 (70.97) | <0.01** |
| PBDE154, pg/g | 3.50 (3.95) | 3.27 (3.54) | 3.82 (4.44) | <0.01** |
| PBDE209, pg/g | 19.91 (30.26) | 19.36 (21.61) | 20.69 (39.29) | 0.03* |
| PBB153, pg/g | 30.66 (60.87) | 24.76 (52.94) | 38.93 (69.68) | <0.01** |

Data are presented as median (IQR). **p* < 0.05, ***p* < 0.01.

**Table S4. Subgroups analysis for the associations between log-transformed serum BFRs and hypertension.**

| Gender | Male | | | Female | | | *P-int* |  |
| --- | --- | --- | --- | --- | --- | --- | --- | --- |
|  | OR | CI | *P* value | OR | CI | *P* value |  |  |
| lgPBDE28 | 1.22 | 0.82-1.81 | 0.32 | 1.17 | 0.74-1.85 | 0.51 | 0.20 |  |
| lgPBDE47 | 1.10 | 0.80-1.51 | 0.56 | 1.09 | 0.75-1.58 | 0.65 | 0.33 |  |
| lgPBDE85 | 1.19 | 0.89-1.59 | 0.24 | 1.06 | 0.77-1.47 | 0.71 | 0.66 |  |
| lgPBDE99 | 1.02 | 0.78-1.34 | 0.90 | 1.00 | 0.72-1.37 | 0.98 | 0.37 |  |
| lgPBDE100 | 1.45 | 1.06-1.98 | 0.02* | 1.06 | 0.76-1.47 | 0.74 | 0.74 |  |
| lgPBDE153 | 1.62 | 1.19-2.21 | <0.01** | 1.32 | 0.94-1.85 | 0.12 | 0.22 |  |
| lgPBDE154 | 1.32 | 0.98-1.78 | 0.07 | 0.94 | 0.68-1.31 | 0.73 | 0.79 |  |
| lgPBDE209 | 0.89 | 0.60-1.33 | 0.58 | 1.16 | 0.78-1.73 | 0.48 | 0.32 |  |
| lgPBB153 | 1.24 | 0.99-1.55 | 0.06 | 0.78 | 0.58-1.04 | 0.09 | 0.93 |  |
| Education | Below college | | | College | | | *P-int* |  |
|  | OR | CI | *P* value | OR | CI | *P* value |  |  |
| lgPBDE28 | 1.19 | 0.77-1.84 | 0.45 | 1.29 | 0.86-1.94 | 0.22 | 0.61 |  |
| lgPBDE47 | 1.09 | 0.75-1.59 | 0.65 | 1.18 | 0.85-1.64 | 0.33 | 0.45 |  |
| lgPBDE85 | 1.01 | 0.73-1.39 | 0.97 | 1.27 | 0.97-1.66 | 0.09 | 0.14 |  |
| lgPBDE99 | 0.97 | 0.71-1.33 | 0.87 | 1.10 | 0.83-1.45 | 0.52 | 0.30 |  |
| lgPBDE100 | 1.24 | 0.87-1.76 | 0.24 | 1.31 | 0.97-1.76 | 0.08 | 0.45 |  |
| lgPBDE153 | 1.62 | 1.17-2.22 | <0.01** | 1.45 | 1.08-1.95 | 0.02* | 0.90 |  |
| lgPBDE154 | 1.03 | 0.72-1.46 | 0.89 | 1.25 | 0.94-1.65 | 0.13 | 0.18 |  |
| lgPBDE209 | 1.38 | 0.91-2.09 | 0.14 | 0.84 | 0.54-1.31 | 0.44 | 0.70 |  |
| lgPBB153 | 1.17 | 0.91-1.5 | 0.23 | 0.97 | 0.79-1.19 | 0.76 | 0.23 |  |
| Ratio of family income  to poverty | <1 | | | >=1 | | | *P-int* |  |
|  | OR | CI | *P* value | OR | CI | *P* value |  |  |
| lgPBDE28 | 1.32 | 0.67-2.60 | 0.42 | 1.24 | 0.9-1.72 | 0.19 | 0.91 |  |
| lgPBDE47 | 1.31 | 0.72-2.37 | 0.38 | 1.13 | 0.88-1.45 | 0.36 | 0.72 |  |
| lgPBDE85 | 1.31 | 0.81-2.13 | 0.28 | 1.13 | 0.91-1.41 | 0.28 | 0.75 |  |
| lgPBDE99 | 1.22 | 0.75-2.01 | 0.42 | 1.03 | 0.82-1.28 | 0.82 | 0.59 |  |
| lgPBDE100 | 1.55 | 0.93-2.58 | 0.10 | 1.24 | 0.98-1.58 | 0.08 | 0.57 |  |
| lgPBDE153 | 1.61 | 0.99-2.61 | 0.05* | 1.47 | 1.17-1.86 | <0.01** | 0.47 |  |
| lgPBDE154 | 1.52 | 0.92-2.52 | 0.11 | 1.11 | 0.87-1.42 | 0.40 | 0.41 |  |
| lgPBDE209 | 1.05 | 0.56-1.96 | 0.89 | 1.03 | 0.73-1.44 | 0.89 | 0.95 |  |
| lgPBB153 | 1.05 | 0.70-1.57 | 0.82 | 1.02 | 0.85-1.23 | 0.84 | 0.20 |  |
| Serum cotinine | | <=1 μg/L | | | >1μg/L | | | *P-int* |
|  |  | OR | CI | *P* value | OR | CI | *P* value |  |
| lgPBDE28 | | 1.08 | 0.74-1.59 | 0.69 | 1.81 | 1.04-3.12 | 0.04* | 0.71 |
| lgPBDE47 | | 1.03 | 0.76-1.40 | 0.84 | 1.43 | 0.92-2.23 | 0.12 | 0.87 |
| lgPBDE85 | | 1.07 | 0.84-1.36 | 0.61 | 1.36 | 0.94-1.96 | 0.10 | 0.75 |
| lgPBDE99 | | 0.96 | 0.75-1.24 | 0.76 | 1.24 | 0.86-1.78 | 0.25 | 0.88 |
| lgPBDE100 | | 1.12 | 0.85-1.48 | 0.43 | 1.73 | 1.14-2.62 | 0.01* | 0.45 |
| lgPBDE153 | | 1.40 | 1.06-1.85 | 0.02* | 1.86 | 1.20-2.88 | 0.01* | 0.25 |
| lgPBDE154 | | 1.00 | 0.77-1.30 | 0.99 | 1.56 | 1.05-2.31 | 0.03* | 0.40 |
| lgPBDE209 | | 0.98 | 0.67-1.44 | 0.91 | 1.19 | 0.74-1.92 | 0.46 | 0.80 |
| lgPBB153 | | 0.97 | 0.79-1.19 | 0.78 | 1.24 | 0.87-1.78 | 0.24 | 0.93 |
| Alcohol consumption | <12 drinks | | | >= 12 drinks | | | *P-int* |  |
|  | OR | CI | *P* value | OR | CI | *P* value |  |  |
| lgPBDE28 | 1.14 | 0.78-1.67 | 0.50 | 1.41 | 0.97-2.06 | 0.08 | 0.70 |  |
| lgPBDE47 | 0.98 | 0.72-1.33 | 0.89 | 1.39 | 1.03-1.88 | 0.04 | 0.21 |  |
| lgPBDE85 | 1.04 | 0.80-1.35 | 0.76 | 1.30 | 0.98-1.72 | 0.07 | 0.50 |  |
| lgPBDE99 | 0.93 | 0.72-1.21 | 0.60 | 1.19 | 0.91-1.56 | 0.21 | 0.35 |  |
| lgPBDE100 | 1.12 | 0.84-1.49 | 0.43 | 1.48 | 1.06-2.07 | 0.02 | 0.36 |  |
| lgPBDE153 | 1.41 | 1.07-1.86 | 0.02* | 1.60 | 1.14-2.25 | 0.01 | 0.64 |  |
| lgPBDE154 | 1.04 | 0.78-1.38 | 0.81 | 1.30 | 0.95-1.77 | 0.10 | 0.52 |  |
| lgPBDE209 | 1.05 | 0.74-1.49 | 0.77 | 1.02 | 0.61-1.73 | 0.93 | 0.97 |  |
| lgPBB153 | 0.92 | 0.73-1.17 | 0.50 | 1.17 | 0.92-1.50 | 0.21 | 0.56 |  |
| Sleep disorders | No | | | Yes | | | *P-int* |  |
|  | OR | CI | *P* value | OR | CI | *P* value |  |  |
| lgPBDE28 | 1.12 | 0.8-1.58 | 0.51 | 1.49 | 0.84-2.64 | 0.17 | 0.82 |  |
| lgPBDE47 | 1.07 | 0.81-1.41 | 0.66 | 1.24 | 0.78-1.96 | 0.37 | 0.70 |  |
| lgPBDE85 | 1.06 | 0.83-1.37 | 0.64 | 1.29 | 0.86-1.94 | 0.22 | 0.41 |  |
| lgPBDE99 | 0.95 | 0.75-1.20 | 0.67 | 1.20 | 0.80-1.82 | 0.38 | 0.35 |  |
| lgPBDE100 | 1.24 | 0.94-1.64 | 0.13 | 1.25 | 0.82-1.91 | 0.29 | 0.96 |  |
| lgPBDE153 | 1.37 | 1.05-1.78 | 0.02* | 1.79 | 1.19-2.69 | 0.01* | 0.29 |  |
| lgPBDE154 | 1.08 | 0.81-1.43 | 0.62 | 1.25 | 0.83-1.87 | 0.29 | 0.58 |  |
| lgPBDE209 | 0.94 | 0.68-1.28 | 0.69 | 1.45 | 0.78-2.69 | 0.24 | 0.16 |  |
| lgPBB153 | 1.07 | 0.86-1.33 | 0.53 | 0.93 | 0.68-1.27 | 0.64 | 0.04* |  |
| Physical Activity | No | | | Yes | | | *P-int* |  |
|  | OR | CI | *P* value | OR | CI | *P* value |  |  |
| lgPBDE28 | 1.22 | 0.72-2.05 | 0.46 | 1.27 | 0.89-1.80 | 0.19 | 0.35 |  |
| lgPBDE47 | 1.09 | 0.72-1.64 | 0.69 | 1.16 | 0.88-1.52 | 0.30 | 0.25 |  |
| lgPBDE85 | 0.97 | 0.67-1.41 | 0.88 | 1.20 | 0.96-1.50 | 0.12 | 0.13 |  |
| lgPBDE99 | 1.01 | 0.71-1.44 | 0.94 | 1.05 | 0.82-1.33 | 0.72 | 0.34 |  |
| lgPBDE100 | 1.10 | 0.72-1.66 | 0.67 | 1.32 | 1.02-1.72 | 0.04* | 0.15 |  |
| lgPBDE153 | 1.99 | 1.22-3.23 | 0.01* | 1.38 | 1.07-1.78 | 0.02* | 0.33 |  |
| lgPBDE154 | 0.98 | 0.66-1.47 | 0.94 | 1.19 | 0.92-1.55 | 0.19 | 0.14 |  |
| lgPBDE209 | 1.20 | 0.69-2.09 | 0.52 | 1.03 | 0.71-1.48 | 0.90 | 0.47 |  |
| lgPBB153 | 0.87 | 0.62-1.23 | 0.44 | 1.06 | 0.87-1.30 | 0.54 | 0.24 |  |
| Diabetes | No | | | Yes | | | *P-int* |  |
|  | OR | CI | *P* value | OR | CI | *P* value |  |  |
| lgPBDE28 | 1.17 | 0.84-1.62 | 0.36 | 1.77 | 0.90-3.50 | 0.10 | 0.73 |  |
| lgPBDE47 | 1.07 | 0.83-1.38 | 0.60 | 1.55 | 0.86-2.80 | 0.15 | 0.86 |  |
| lgPBDE85 | 1.09 | 0.88-1.35 | 0.45 | 1.41 | 0.81-2.46 | 0.23 | 0.80 |  |
| lgPBDE99 | 0.98 | 0.79-1.22 | 0.88 | 1.32 | 0.82-2.11 | 0.25 | 0.76 |  |
| lgPBDE100 | 1.19 | 0.93-1.51 | 0.16 | 1.69 | 0.95-3.02 | 0.08 | 0.62 |  |
| lgPBDE153 | 1.39 | 1.09-1.78 | 0.01* | 2.33 | 1.38-3.93 | <0.01** | 0.05 |  |
| lgPBDE154 | 1.06 | 0.83-1.35 | 0.63 | 1.67 | 0.94-2.97 | 0.08 | 0.56 |  |
| lgPBDE209 | 0.98 | 0.71-1.35 | 0.89 | 1.44 | 0.57-3.65 | 0.45 | 0.77 |  |
| lgPBB153 | 1.00 | 0.82-1.23 | 0.96 | 1.18 | 0.67-2.09 | 0.56 | 0.50 |  |

Analyses were adjusted for age, gender, race, education level, PIR, serum cotinine, alcohol consumption, sleep disorders, depression, physical activity, dietary sodium intake, dietary potassium intake, BMI, eGFR, history of diabetes and NHANES cycles. **p* < 0.05, ***p* < 0.01.

**
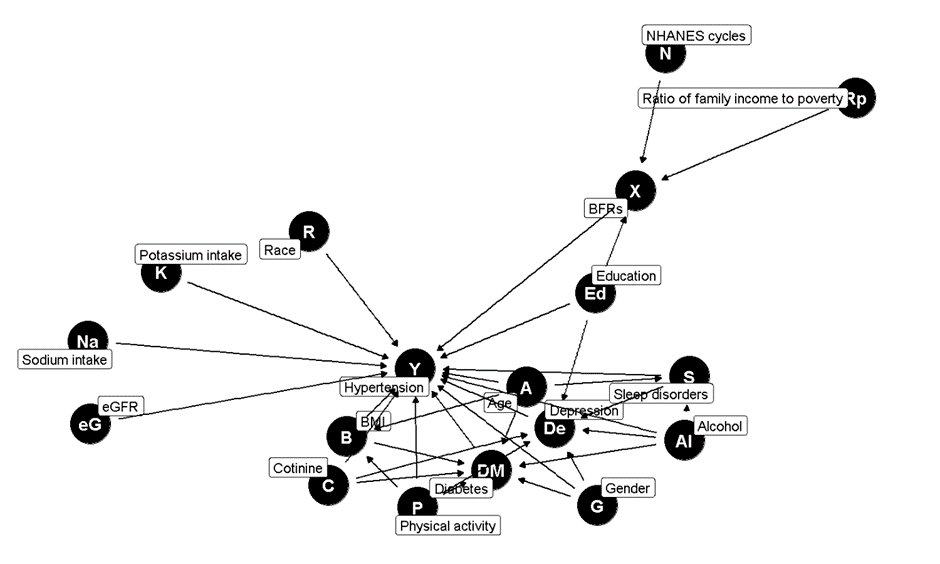
**

**Fig. S1.** DAG


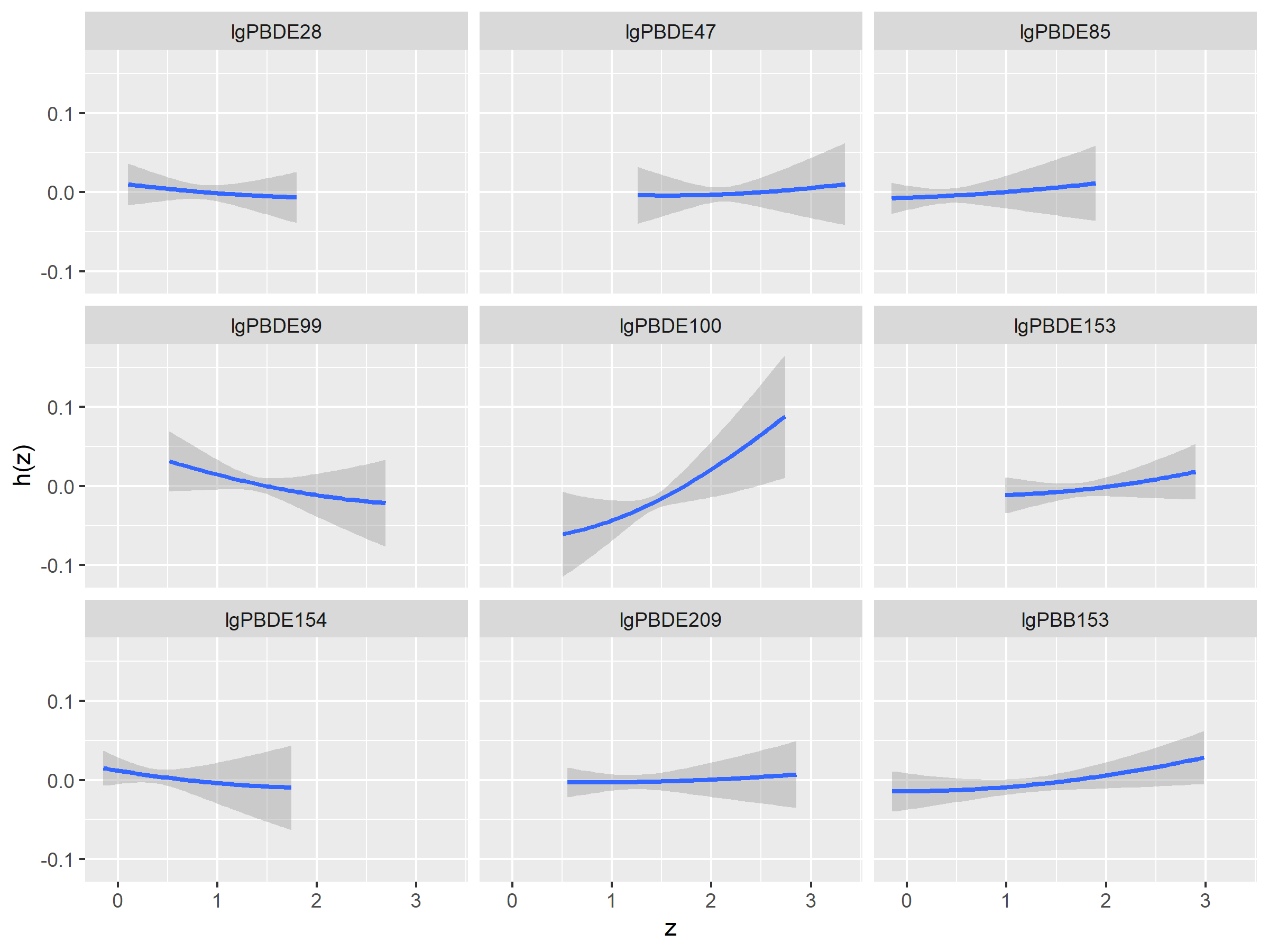


**Fig. S2.** Univariate exposure-response functions (95%CrI) for BFRs in hypertension

Univariate exposure–response functions and 95% confidence interval for each BFR with the other 8 BFRs fixed at the median.


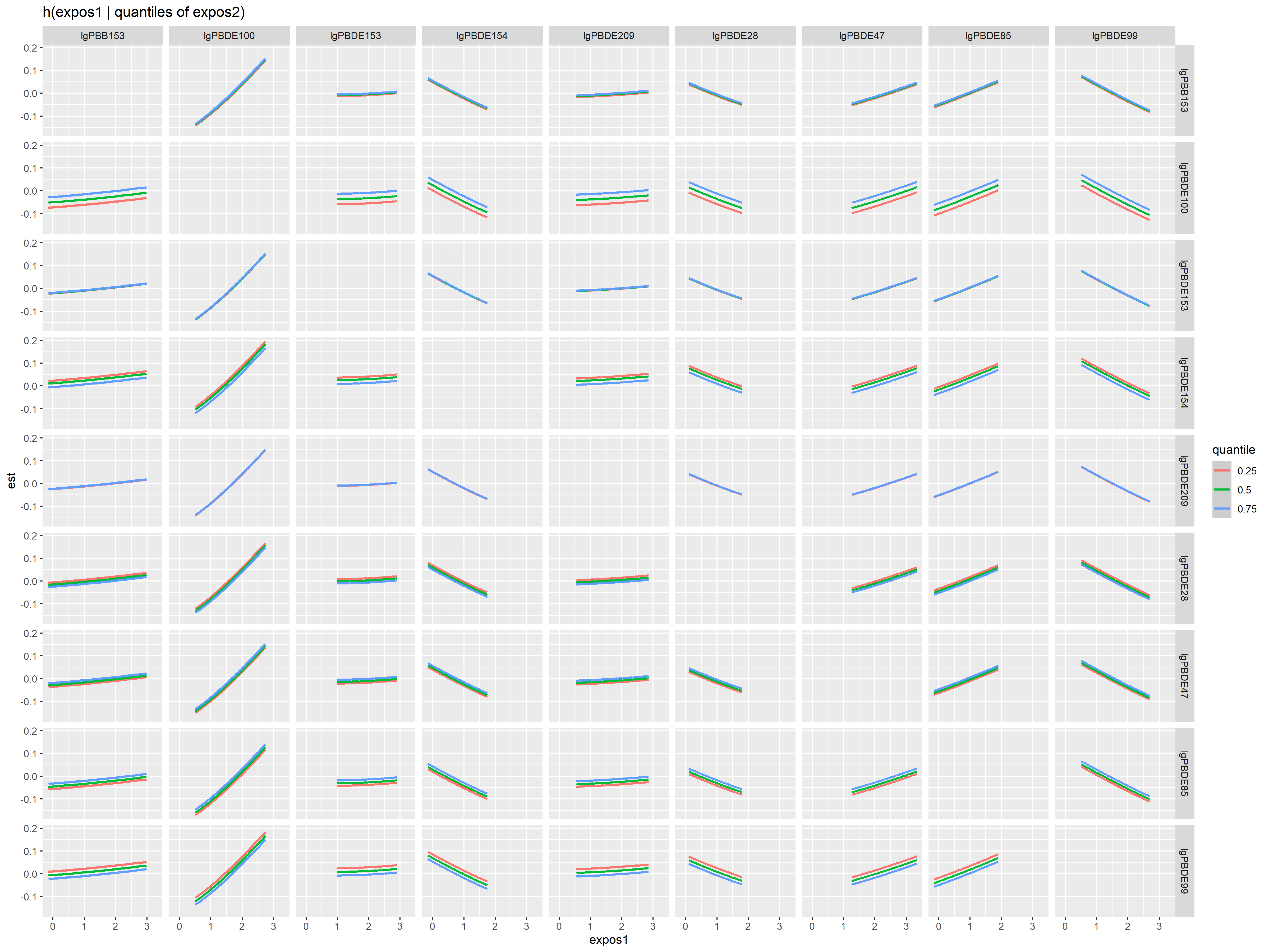


**Fig. S3.** The interaction effects of BFRs on hypertension.
